# De Novo Heart Failure in a 32-Year-Old Man From Congenital Ostial Left Main Coronary Atresia

**DOI:** 10.1016/j.cjcpc.2025.12.001

**Published:** 2025-12-17

**Authors:** Gary Wang, Taylor Petropoulos, Osami Honjo, Rafael Alonso-Gonzalez, Adriana Luk, Filio Billia

**Affiliations:** a Division of Cardiology, Department of Medicine, University of Ottawa Heart Institute, Ottawa, Ontario, Canada; b Peter Munk Cardiac Center, University Health Network, Toronto, Ontario, Canada; c Division of Cardiovascular Surgery, Labatt Family Heart Centre, Hospital for SickKids, Toronto, Ontario, Canada

**Keywords:** congenital, left main coronary artery ostial atresia, heart failure, cardiomyopathy

**A previously healthy 32-year-old man presented with 2 weeks of progressive abdominal bloating, exertional dyspnea, and chest discomfort relieved by rest. His history was unremarkable, without cardiovascular risk factors, prodromal illness, or substance use. On arrival, he was markedly hypertensive and tachycardic, with bibasilar crackles but no peripheral congestion. Initial investigations revealed mild renal dysfunction, markedly elevated N-terminal pro–b-type natriuretic peptide, minimally elevated troponin, and nonspecific electrocardiogram abnormalities. Chest radiography showed only subtle vascular changes. Despite medical stabilization, early cardiac imaging demonstrated severe biventricular dysfunction, prompting an extensive diagnostic evaluation to clarify the etiology of his new-onset heart failure.**

## History of Presentation

A 32-year-old man presented with a 2-week history of worsening abdominal bloating, dyspnea, and chest pain on exertion, which improved with rest. He was previously active without any functional limitations at any point before this. There was no viral prodrome, recent chest trauma, or risk factors for thrombosis. The patient had no family history, comorbidities, or allergies. He was a lifelong nonsmoker and a social drinker. He denied illicit drug use.

Admission vital signs included a blood pressure (BP) of 193/138 mm Hg, a heart rate of 120 beats per minute, a respiratory rate of 20 breaths per minute, and an O_2_ saturation of 97% on room air. There was no BP differential between arms. He was afebrile. On examination, he had normal heart sounds with no evidence of peripheral congestion, although bilateral respiratory crackles in the lower lung zones were evident.

**Figure 1:**
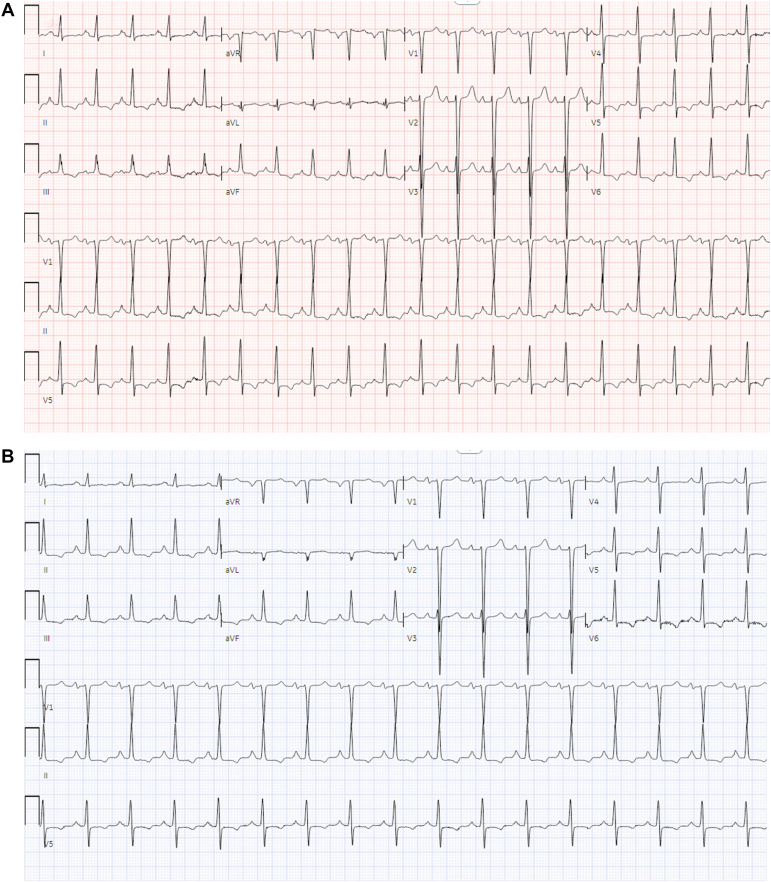
Electrocardiograms (**A**) at presentation showing sinus tachycardia with right atrial enlargement, QRS 88 milliseconds, 1-mm ST elevations in V2-V3, and ST depressions seen in the inferolateral leads, and (**B**) within 24 hours of presentation showing sinus rhythm with QRS 91 milliseconds and diffuse T-wave abnormalities.

Initial laboratory findings showed normal complete blood count and electrolytes, elevated creatinine at 112 μmol/L (normal: 53-106 μmol/L), N-terminal pro–b-type natriuretic peptide of 6833 ng/L (normal: <300 ng/L), and high-sensitivity troponin at 34 ng/L (normal: ≤26 ng/L), which was 30 ng/mL when repeated. The chest radiograph showed mild vascular prominence and mild thickening of the fissures, but no pulmonary edema nor pleural effusions (Supplemental Fig. S1). The initial electrocardiogram showed sinus tachycardia with right atrial enlargement, a QRS duration of 88 milliseconds, 1-mm ST elevations in V2-V3, and ST depressions seen in the inferolateral leads (Fig. 1A).

He was admitted to the coronary intensive care unit and initiated on sodium nitroprusside, which improved his symptoms of abdominal bloating and chest pain and stabilized his heart rate (Fig. 1B). An echocardiogram showed a mildly dilated left ventricle (LV) (left ventricular internal diameter in diastole 64 mm) with left ventricular ejection fraction (LVEF) 18%, moderately reduced right ventricular systolic function, biatrial dilatation, and right ventricular systolic pressure estimated at 46 mm Hg (right atrial pressure 8 mm Hg). A broad differential diagnosis for the reduced LVEF was discussed, including hypertension and ischemic and nonischemic cardiomyopathy (eg, viral, infiltrative, toxin-induced cardiomyopathy, and Takotsubo). His elevated BP at presentation made hypertensive cardiomyopathy our leading differential diagnosis. We tested for secondary causes of hypertension, but found none (see Supplementary Table S1). Interestingly, he had an initial urine albumin:creatinine ratio that was elevated at 30.5 mg/mmol, which was deemed to be likely due to his hypertension. Furthermore, he had normal pulmonary function tests and no evidence of exogenous steroid usage. Ultrasound, pyrophosphate, and computed tomography (CT) imaging did not show evidence of renal artery stenosis or adrenal nodules. Genetic testing was performed, which indicated that our patient had a pathogenic titin (TTN) mutation (p.Arg26628∗), with results available 3 months after discharge.

He was transitioned to oral guideline-directed medical therapy (metoprolol 25 mg orally twice daily, perindopril 12 mg orally daily, spironolactone 50 mg orally daily, amlodipine 10 mg orally twice daily), which also controlled the BP in a timely fashion. Repeat transthoracic echocardiography showed an LVEF of 22% with grade 2 diastolic dysfunction. A cardiac magnetic resonance imaging (MRI) corroborated the impaired global systolic LV function with an EF of 14%, concentric left ventricular hypertrophy with subtle diffuse interstitial changes, and no ischemic type changes.

**Figure 2:**
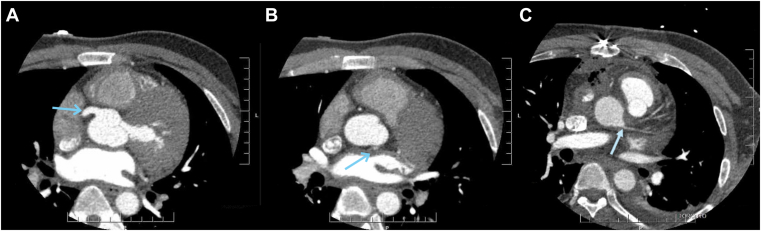
Coronary CT showing (**A**) ostium of a dominant RCA (**arrow**) emerging from the right coronary cusp. (**B**) Left coronary cusp with no distinct lumen connected to the left main (**arrow**). The left main coronary artery is small in caliber. (**C**) CT coronary performed after left main ostioplasty demonstrating connection of the left main coronary artery to the aorta (**arrow**). CT, computed tomography; RCA, right coronary artery.

Without a satisfactory diagnosis a week into the admission, we decided to perform chest CT with contrast, which showed possible ostial atresia of the left main (LM). Also noted were small bilateral pleural effusions, pulmonary edema, and enlarged left heart chambers. A subsequent cardiac CT showed a calcium score of 46, and the LM appeared to have a truncated origin from the left aortic sinus and was supplied by retrograde flow from an enlarged right coronary artery (Fig. 2, A and B). Coronary angiography confirmed a sole right dominant system with the left system supplied by the right coronary artery at the level of the LM. Pullback across the aortic valve demonstrated no important transvalvular gradient. A dobutamine stress echocardiogram before discharge showed a baseline stroke volume of 25 mL (11.9 mL/m^2^), with a peak at 57 mL (27.1 mL/m^2^), suggesting contractile reserve.

## Management and Follow-up

Multidisciplinary discussions took place with the working diagnosis including congenital atresia of the left coronary ostium, on the background of a possible nonischemic cardiomyopathy based on his cardiac MRI findings. He was discharged from hospital in stable condition with acetylsalicylic acid added to his medical therapy. At the time of discharge, he had a repeat echocardiogram showing an LVEF of 30%.

An outpatient perfusion positron emission tomography with rubidium demonstrated a large, moderate intensity defect in the anterolateral wall associated with a wall motion abnormality suggestive of significant ischemia in the left anterior descending and circumflex territories. On the basis of these findings, he underwent ostioplasty of the LM trunk. Intraoperatively, the left main coronary artery was located outside of the aortic wall adjacent to the commissure between the left and right coronary cusp, with no connection between the left coronary artery and the aorta. The left coronary artery was fully mobilized from the aortic root. The hypoplastic left main segment was enlarged with an autologous pulmonary artery patch, which was procured from the patient’s main pulmonary artery. A large round-shaped opening was created in the middle of the left coronary sinus, and the left coronary artery was implanted to the aortic root in an end-to-side fashion. The defect on the main pulmonary artery was reconstructed with an autologous pericardial patch. The surgery was uncomplicated, and at the time of discharge, his LVEF recovered to 56% with no regional wall motion abnormalities.

## Discussion

Here we present a case of *de novo* HF with global hypokinesis in a young patient with a complex diagnostic pathway who presented initially with hypertensive crisis. First, the hypertension required a nitroprusside infusion and multiple oral antihypertensive agents placing hypertensive cardiomyopathy at the top of the differential and the workup focusing on secondary causes of hypertension. Additional testing was performed, including cardiac MRI and pyrophosphate scan for infiltrative disorders such as sarcoid and amyloid. Genetic testing for etiologies such as Fabry’s, familial cardiomyopathy, and extensive infectious and autoimmune assessments revealed a pathogenic mutation in titin. Titan mutations occur in 25% of familial and 18% of sporadic cases of nonischemic cardiomyopathy.^1^ However, cardiac CT and subsequent catheterization solidified the diagnosis of LM coronary artery ostial atresia (LMCAOA).

Given the extensive workup, the case here emphasizes the importance of assessing coronary artery disease in young patients and the need for a multidisciplinary team approach. In *de novo* HF cases with a clinical picture suspicious for nonischemic etiology, coronary disease and/or anomalies should be promptly ruled out because LMCAOA is an extremely rare congenital anomaly.

In a recent review of 93 cases, 50 were pediatric patients and the oldest patient was 85 years old at presentation.^2^ Patients presented with a variety of different symptoms, including HF, syncope, chest pain, and cardiac arrest. These symptoms are thought to arise as the myocardial demand eventually surpasses the ability of collaterals to provide adequate perfusion. Revascularization, whether through ostioplasty or coronary artery bypass grafting (CABG), has been found to improve mortality among these patients and remains the gold standard of therapy. Among the adult patients in the review, 15 were medically managed, of whom 10 survived, the 18 who received revascularization all survived, and the outcomes of the remaining 10 are unknown.^2^ Additional case reports of LMCAOA have since been reported, with medical management and CABG being the treatments chosen.^3^^,^^4^

For our patient, the case was reviewed with our congenital cardiac surgeons, who deemed the patient a candidate for coronary ostioplasty with a patch. Although CABG has been the revascularization modality chosen in previous reports cases in adults, in our case there was concern for stenosis and occlusions in bypass grafts due to intimal proliferation and hyperplasia. There remained uncertainty for long-term patency in our young patient, especially because he would require 2 grafts—to both the left anterior descending and circumflex. On the other hand, coronary ostioplasty provides antegrade coronary blood flow. Case series and single-center experiences showed favorable long-term patency and outcomes with ostioplasty in the pediatric and adolescent populations.^5^, ^6^, ^7^

His degree of LV dysfunction increased the surgical risk. Fortunately, the addition of guideline-directed medical therapy led to an improvement in LVEF. A decision was then made to discharge the patient to allow for further recovery and assess for inducible ischemia with a positron emission tomography scan. With evidence of a large territory of ischemia, he then underwent LM ostioplasty (Fig. 2C) with further recovery of the EF. Notably, this is the first adult patient with LMCAOA who was successfully treated with coronary ostioplasty.

Genetic testing revealed a mutation of TTN (p.Arg26628∗) with a known predisposition to nonischemic cardiomyopathy.^1^ In this case, the recovery after revascularization and lack of family history suggest that the HF was less likely from a primary mutation intrinsic to the myocardium. However, the potential association between LMCAOA and TTN mutations remains unclear. This case highlighted a rare congenital coronary anomaly presenting in adulthood that was found in a comprehensive workup including assessment of the coronary anatomy. Furthermore, we outline the considerations regarding revascularization and report the first known case of successful coronary ostioplasty in an adult with LMCAOA.

**Novel Teaching Points**

• In young patients with new-onset heart failure, coronary anomalies must be excluded early—even when the clinical picture initially suggests a nonischemic etiology such as hypertensive or genetic cardiomyopathy. This case highlights how left main coronary artery ostial atresia (LMCAOA), although extremely rare, can present in adulthood and mimic other causes of cardiomyopathy.
• Revascularization strategy in LMCAOA should be individualized, and coronary ostioplasty can be a viable option in selected adults. Although CABG is commonly reported, ostioplasty offers restored antegrade flow and may provide superior long-term patency in young patients, as demonstrated by this first documented adult case.
