# Supplementary material for: De Novo Heart Failure in a 32-Year-Old Man From Congenital Ostial Left Main Coronary Atresia
Source: CJC Pediatr Congenit Heart Dis. 2025 Dec 17;5(3):185–8. doi: 10.1016/j.cjcpc.2025.12.001 (PMC13343408; doi:10.1016/j.cjcpc.2025.12.001)

**Supplemental Table S1:** Initial laboratory work-up and results.

| **Lab Test** | **Result** | **Normal Value** |
| --- | --- | --- |
| Hemoglobin A1C | 5.1% | <=5.9% |
| TSH | 1.479 mIU/L | 0.350 – 4.940 mIU/L |
| Cholesterol | 4.24 mmol/L | <5.2 mmol/L |
| Triglycerides | 2.04 mmol/L | <1.70 mmol/L |
| Non-HDL | 3.63 mmol/L | <3.37 mmol/L |
| LDL | 2.80 mmol/L | <4.2 mmol/L |
| HDL | 0.61 mmol/L | 1.0-1.6 mmol/L |
| Total cholesterol/HDL ratio | 7.0 |  |
| ApoB | 1.51 g/L | 0.66 – 1.44 g/L |
| Lipoprotein (a) | 35.1nmol/L | 0.0 – 100.0 nmol/L |
| Urine Albumin to Creatinine ratio | 30.5 mg/mmol | M= 2.9 mg/mmol |
| ANA | Negative (x2) | Negative |
| Anti-dsDNA | <1 IU/mL | <= 4 IU/mL |
| RF | <7 IU/mL | <= 11 IU/mL |
| Anti-MPO | <0.2 AI units | <= 0.9 AI units |
| Anti-PR3 | <0.2 AI units | <= 0.9 AI units |
| C3 | 1.17 g/L | 0.98 – 1.96 g/L |
| C4 | 0.21 g/L | 0.10 – 0.40 g/L |
| Serum Protein Electrophoresis | No monoclonal protein detected  Total protein 70g/L | 65 – 80 g/L |
| Urine Protein Electrophoresis | Trace amounts of albumin and other proteins present (0.21 g/L) | Absence of protein |
| Renin | 8.7 ng/L | Normal (recumbent > 6hr) = 0.0 – 11.2 ng/L |
| Aldosterone | 244 pmol/L | Normal: 165 – 969 pmol/L (standing), 76 – 523 pmol/L (recumbent) |
| Adrenocorticotropic Hormone | 2.3 pmol/L | <= 10.2 pmol/L |
| Aldosterone/Renin Ratio | 28 pmol/ng | <100 pmol/ng |
| Normetanephrine | 0.49 nmol/L | <0.90 nmol/L |
| Metanephrine | 0.17 nmol/ | <0.50 nmol/L |
| 24h Urine Free Cortisol | 727 nmol/D | 12 – 486 nmol/D |
| 3-Methoxytyramine | <0.05 nmol/L 1 mo ago  <0.05 nmol/L 2 mo ago | <0.30 nmol/L |
| Testosterone | 9.0 nmol/L | 7.7 – 30.2 nmol/L |
| Free + Albumin Bound Testosterone | 2.6 nmol/L | 4.0 – 17.0 nmol/L |
| Dehydroepiandrosterone | 7.4 umol/L | 4.6 – 16.1 umol/L |
| Sex Hormone Binding Globulin | 65 nmol/L | 11 – 78 nmol/L |
| Serum CMV IgG | Negative | Negative |
| Hep B Core Total Ab | Negative | Negative |
| Hep B Surface Ab | <2.00 IU/L | Evidence of Immunity: >= 10 IU/L |
| Hep B Surface Antigen | Negative (<0.02 IU/mL) | Negative (<0.05 IU/mL) |
| Hep C Ab | Negative | Negative |
| EBV VCA IgG Ab (Immunity) | Positive | Negative |
| Toxoplasmosis IgG | Non-reactive | Non-reactive |
| CMV IgG | Negative | Negative |
| HIV 1&2 Antibody/p24 Antigen | Negative | Negative |
| HTLV 1&2 Antibody | Negative | Negative |
| Measles IgG | Non-reactive | Non-reactive |
| Varicella Zoster IgG | Positive | Negative |
| Treponema Pallidum Antibody IgG + IgM | Negative | Negative |

**Supplemental Figure S1:** PA (left) and lateral (right) chest xray micrographs at the time of presentation showing evidence of mild vascular prominence and mild thickening of the fissures, but no pulmonary edema nor pleural effusions.


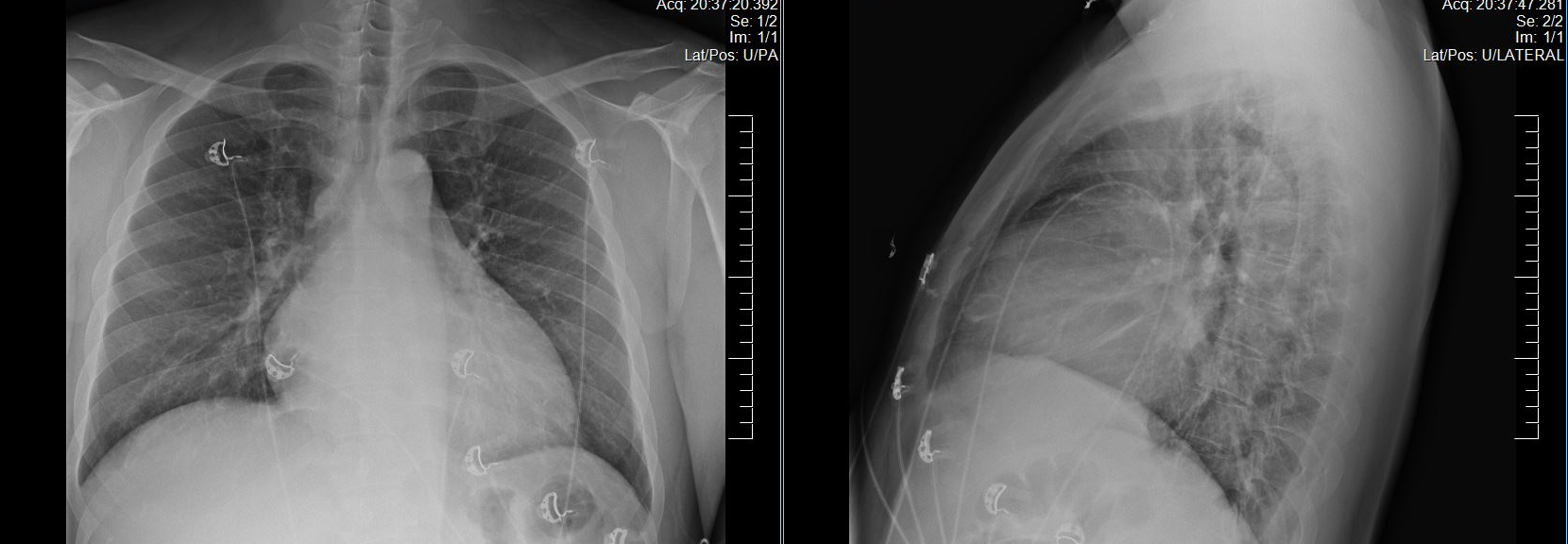

Supplement: Supplementary — Table [file mmc1.docx]
